# Single‐Cell RNA Seq in Sydenham Chorea Shows B Cell *HLA‐DR/DQ* Upregulation and Plasma Cell Proteasomal Activation

**DOI:** 10.1002/acn3.70179

**Published:** 2025-08-26

**Authors:** Velda X. Han, Brooke A. Keating, Brian S. Gloss, Xianzhong Lau, Ruwani Dissanayake, Hiroya Nishida, Jessica Hayes, Shrujna Patel, Shekeeb S. Mohammad, Russell C. Dale

**Affiliations:** ^1^ Kids Neuroscience Centre, The Children's Hospital at Westmead, Faculty of Medicine and Health University of Sydney Sydney New South Wales Australia; ^2^ Khoo Teck Puat‐National University Children's Medical Institute, National University Health System Singapore Singapore; ^3^ Department of Paediatrics, Yong Loo Lin School of Medicine National University of Singapore Singapore Singapore; ^4^ Westmead Research Hub, Westmead Institute for Medical Research Westmead New South Wales Australia; ^5^ Australian Genome Research Facility Ltd Melbourne Victoria Australia; ^6^ Australian Genome Research Facility Ltd Westmead New South Wales Australia; ^7^ The Children's Hospital at Westmead, Faculty of Medicine and Health University of Sydney Sydney New South Wales Australia; ^8^ The University of Sydney, Faculty of Medicine and Health, School of Medical Sciences and Discipline of Child and Adolescent Health Sydney New South Wales Australia

**Keywords:** autoimmune brain disorder, neuroimmunology, neuropsychiatric, transcriptomics

## Abstract

The pathogenesis of Sydenham chorea remains unclear. We report a 10‐year‐old girl presenting with subacute chorea and mild carditis following Streptococcal throat infection. Single‐cell RNA sequencing on 30,794 peripheral immune cells from the patient and two sex‐matched controls revealed nine immune cell clusters. We found up‐regulated antigen processing pathways in B cells, monocytes, and eosinophils, enriched by *HLA* genes, particularly *HLA‐DRB5* and *HLA‐DQB1*, and immunoglobulin (*IGH*) genes in B cells. Up‐regulated proteasomal pathways in plasma cells were enriched by *PSM* genes. These insights support the B cell‐mediated autoantibody hypothesis in Sydenham chorea. We highlight the potential of scRNA‐seq in rare diseases to inform targeted therapies.

## 1 Introduction

Globally, an estimated 470,000 new cases of acute rheumatic fever occur each year, contributing to a significant disease burden [1]. Sydenham chorea (SC) is a neurological manifestation of acute rheumatic fever, typically arising weeks to months after a group A streptococcal (GAS) infection. Core symptoms include chorea, hypotonia, and emotional and psychiatric disturbances [2]. Rheumatic fever is widely accepted as a post‐streptococcal autoimmune disease, thought to be predominantly mediated by T cells, with *HLA* vulnerability [3, 4]. However, the exact pathophysiology of SC remains incompletely understood. The leading hypothesis is an autoimmune response triggered by molecular mimicry, where antibodies generated against GAS antigens cross‐react with neurons in the basal ganglia. To date, however, no definite pathogenic autoantibody has been defined that has been translated into clinical practice [5].

Treatment for SC includes corticosteroids and intravenous immunoglobulin, yet 20%–40% experience persistent neuropsychiatric symptoms [2]. No other targeted therapies have been established. Relapses occur in up to 20% of cases, and long‐term intramuscular penicillin prophylaxis is used to prevent recurrence, predominantly to protect the heart [2]. Here, we report an adolescent diagnosed with SC and perform single‐cell RNA sequencing on peripheral immune cells to explore underlying disease mechanisms.

## 2 Methods

### 2.1 Patient and Controls

A prepubertal 10‐year‐old female with 2 weeks of increasing chorea was recruited after informed consent and before immune or antibiotic treatment. Two gender‐matched healthy controls (a prepubertal 12‐year old female and postpubertal 17‐year‐old female without neurological/immune disorders) were also recruited.

### 2.2 Single‐Cell RNA Sequencing—Bioinformatic and Enrichment Analysis

Single‐cell RNA sequencing (scRNA‐seq), using the HIVE platform, was performed (Methods S1). ScRNA‐seq data were analyzed in the R statistical environment [6] with *tidyverse* [7], described in Methods S1. For HIVE scRNA‐seq, the *Seurat* package was used for analysis [8]. Pathway enrichment analysis was performed via Gene Set Enrichment Analysis (GSEA) to obtain enriched Gene Ontology (GO) pathways (FDR < 0.05) using the *clusterProfiler* [9] package (Methods S1).

### 2.3 Ethics Statement

Ethical approval was granted by the Sydney Children's Hospitals Network Human Research Ethics Committee (HREC/18/SCHN/227, 2021/ETH00356).

## 3 Results

### 3.1 Clinical Presentation

Our patient is a 10‐year‐old white Australian girl, with no known Aboriginal or Torres Strait Islander background. She initially presented with a sore throat, clinically diagnosed with tonsillitis (no throat culture performed) and treated with a course of amoxicillin. Over the following 2 weeks, she developed progressive generalized chorea, neuropsychiatric symptoms, and mild carditis. Her mother has antiphospholipid syndrome presenting with recurrent pregnancy loss (two children from six pregnancies), managed with heparin in pregnancy, but no other thrombotic complications. Her father has vitiligo.

Our patient had an ongoing sore throat during her presentation to neurology but no arthralgia or subcutaneous nodules. Examination showed some emotional lability and generalized chorea; the left side of her body was more affected than the right. Her Sydenham chorea rating score was 56 using the UFMG Sydenham's chorea Rating scale (USCRS), indicating moderate chorea, but she was still able to walk and do most self‐care. Her magnetic resonance imaging and angiogram of the brain were normal, antistreptolysin O titre (ASOT) was elevated at 894 IU/mL, throat culture was negative, PR interval was normal on electrocardiogram, and echocardiogram showed mild mitral regurgitation. Her lupus autoantibodies and antiphospholipid antibodies were negative. She received a 3‐day course of intravenous (IV) methylprednisolone (30 mg/kg). Her chorea and neuropsychiatric symptoms resolved after 2.5 weeks post steroid treatment (USCRS Score 2), without further relapses at 12 months of follow‐up. She is continued on monthly intramuscular (IM) penicillin and has ongoing mild mitral regurgitation.

### 3.2 Single‐Cell RNA Sequencing (scRNA‐Seq)

A total of 30,794 cells were sequenced across three samples (one patient and two controls). Uniform manifold approximation and projection (UMAP) analysis of samples revealed nine distinct cell clusters (Figure S1). Differentially expressed genes (DEGs) with FDR < 0.05 ranged from 2 to 1164 DEGs per cell type in SC patient vs. control. GSEA analysis using a ranked gene list derived enriched GO pathways (FDR < 0.05) for individual cell types. The most significant DEG in B cells was *HLA‐DRB5* (log fold change 1.38, adjusted *p* value 2.8 × 10^−42^) which was also the most significant DEG in classical monocytes (log fold change 1.99, adjusted *p* value 8.7 × 10^−164^).

### 3.3 Enriched Pathways Across Cell Types

**FIGURE 1:**
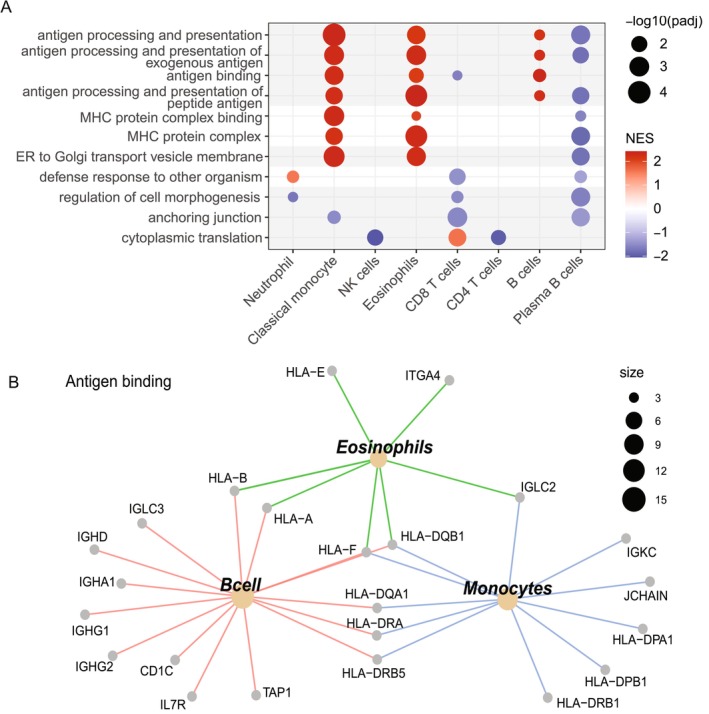
(A) Dot plot of top 5 up‐ and down‐regulated GSEA GO pathways across cell types in SC patient vs. control. Significant pathways (FDR < 0.05) were simplified [9], and only those that were present in > 2 cell types were plotted. In SC patient vs. control, antigen processing, MHC protein complex pathways were up‐regulated in classical monocytes, eosinophils, and B cells, and down‐regulated in plasma B cells. Other innate immune pathways (‘defence response to other organisms’) were up‐regulated in neutrophils, but down‐regulated in CD8 T cells and plasma B cells. (B) Connectivity network plot of the up‐regulated ‘antigen binding’ pathway in classical monocytes, eosinophils, and B cells was plotted. The antigen binding pathways were enriched by *HLA* genes (*HLA‐DR* and *HLA‐DQ* genes) across cell types and immunoglobulin heavy and light chain (*IGH*, *IGL* genes) in B cells.

The top 5 up‐regulated and down‐regulated GSEA GO pathways of individual cell types in SC patient versus control showed similarities and differences in pathways across cell types (dot plot in Figure 1A, all cell types in Figure S2). In SC patient vs. control, antigen processing and MHC protein complex pathways were up‐regulated in classical monocytes, eosinophils, B cells, and down‐regulated in plasma B cells and CD8 T cells. Other innate immune pathways were up‐regulated in neutrophils (defence response to other organism), but down‐regulated in CD8 T cells and plasma B cells.

Connectivity network plot of the up‐regulated ‘antigen binding’ pathway in the classical monocytes, eosinophils, and B cells was plotted (Figure 1B). The antigen binding pathways were enriched by *HLA* genes (*HLA‐DR* and *HLA‐DQ* genes across cell types) and immunoglobulin heavy and light chain (*IGH*, *IGL*) genes (in B cells).

### 3.4 Top 5 Up and Down‐Regulated Pathways in Plasma B Cells

**FIGURE 2:**
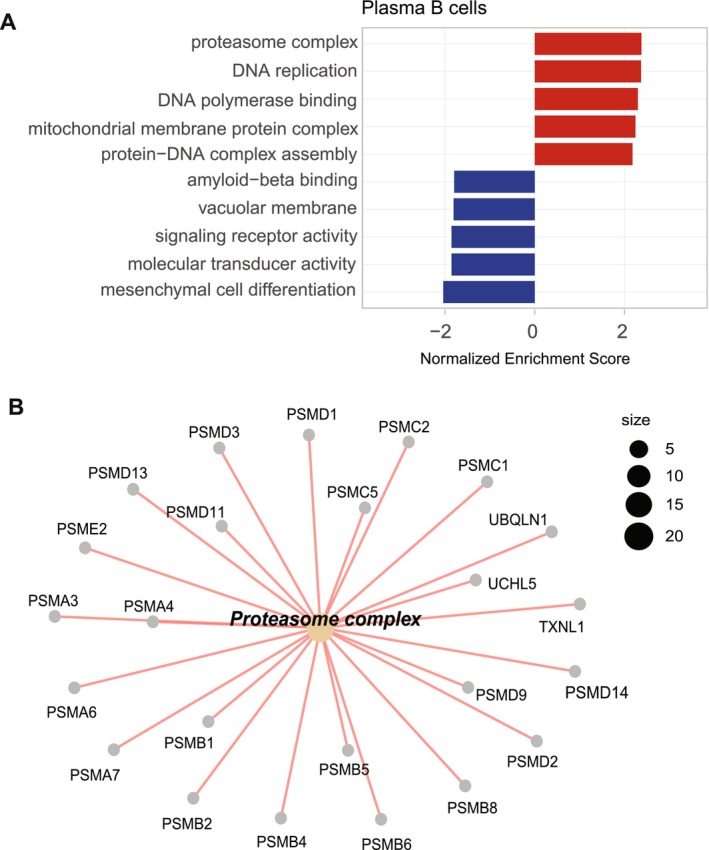
(A) Bar chart of top 5 up‐ and down‐regulated Gene Set Enrichment Analysis (GSEA) Gene Ontology (GO) pathways in plasma B cells. (B) Connectivity network plot of the up‐regulated ‘proteasomal complex’ pathway in plasma B cells was plotted, enriched by *PSM* genes, which are proteasomal structural and regulatory genes.

In the SC patient vs. control, top 5 up‐regulated GO pathways (in red) in plasma B cells included proteasome complex, DNA processing (DNA replication, DNA polymerase binding, protein‐DNA complex assembly), and mitochondrial membrane protein complex (Figure 2A).

Connectivity network plot of the up‐regulated ‘proteasomal complex’ pathway in plasma B cells was plotted, enriched by *PSM* genes, which are proteasomal structural and regulatory genes (Figure 2B).

## 4 Discussion

In this adolescent with SC and mild carditis following Streptococcal throat infection, scRNA‐seq on peripheral immune cells revealed widespread activation of antigen processing pathways across multiple immune cell types, enriched by *HLA‐DR* and *HLA‐DQ* genes, and immunoglobulin genes (in B cells). Plasma B cells showed marked upregulation of the proteasomal pathway, enriched by *PSM* genes which are core and regulatory subunits of the proteasome complex. These findings provide evidence of a B cell‐mediated autoimmune pathogenesis in SC and provide further insights into the underlying immunopathogenic mechanisms of SC.

In our study, broad enrichment of antigen presentation pathways across cell types was primarily driven by *HLA‐DR* and *HLA‐DQ* (e.g., *HLA‐DQB1*) genes, and *HLA‐DRB5* was also the most significant DEG in B cells and classical monocytes. Meta‐analyses indicate that specific HLA class II alleles, including HLA‐DRB1*07 and HLA‐DQA1‐DQB1 (susceptibility haplotypes), versus HLA‐DRB1*15 (a protective allele), confer genetic predisposition to manifestations of rheumatic fever, including rheumatic heart disease and SC [3, 4]. Cross‐reactive epitopes exhibit higher binding affinity to HLA class II risk haplotypes, supporting molecular mimicry as a central mechanism in RHD and SC [4]. Different HLA class II alleles have been linked to different rheumatic fever manifestations, suggesting that specific immune responses shape disease phenotype [10, 11]. Further study into cell‐specific HLA epitope expression is needed to clarify disease susceptibility, and tissue‐specific immune responses in SC. Interestingly, although SC and paediatric autoimmune neuropsychiatric disorders associated with Streptococcal infections (PANDAS) have been linked due to a possible shared streptococcal trigger, a recent study found no HLA class II association in PANS [12]. Additionally, upregulation of immunoglobulin genes in B cells, which encode antibodies, further supports the autoimmunity hypothesis for SC. Dysregulation or faulty selection of B cells expressing immunoglobulin genes can result in the production of autoreactive antibodies, contributing to inflammation and tissue damage in the context of SC.

The ubiquitin‐proteasomal pathway is a key cellular mechanism for degrading and recycling intracellular proteins [13], which regulates essential processes including cell cycle progress, apoptosis, cell trafficking, and modulation of immune and inflammatory responses [13]. This system targets damaged, oxidised, or misfolded proteins as well as regulatory proteins critical to cellular functions [13]. Dysregulation of this pathway has been implicated in the pathogenesis of neurodegenerative, inflammatory, and autoimmune diseases [13, 14]. In our patient, many of the upregulated proteasomal genes are known to be inducible by interferons and contribute to the formation of immunoproteasome. In immune cells, the immunoproteasome processes intracellular proteins into peptides for antigen presentation on MHC class I molecules, essential for CD8+ T cell‐mediated immunity [15, 16]. Increased proteasomal activity in plasma cells may reflect heightened antigen processing and immune activation. These findings highlight the need for further investigations into the role of the proteasomal pathway in SC, including the potential therapeutic relevance of proteasome inhibitors in autoimmune disease [17].

This study presents the first scRNA‐seq analysis of peripheral immune cells in SC. Using scRNA‐seq, we identified key cell type‐specific pathways driving SC pathogenesis, highlighting its value in examining immune mechanisms even in an *n* = 1 context [18]. Further investigations into these pathways are essential to identify effective therapies and prevent disease relapses. Limitations of this report include the examination of a single case, analysis of blood rather than cerebrospinal fluid, and the lack of a post treatment sample. HLA typing was not performed in our patient. Future studies should include serum and cerebrospinal fluid examination, omics analysis, and functional immunology assessment, pre and post treatment, in larger patient cohorts with appropriate controls to minimise confounders (such as the effects of puberty). Additionally, future studies could include controls with uncomplicated GAS pharyngitis to determine if immune responses are specific to SC or reflect GAS infection alone.

## 5 Conclusion

Single‐cell RNA sequencing revealed broad activation of antigen processing and plasma B cell proteasomal pathways in SC, suggestive of a B‐cell mediated autoimmune pathogenesis. Further evaluation of these pathways will be crucial for formulating therapeutic strategies for SC. This case highlights the potential of scRNA sequencing in understanding disease mechanisms in rare diseases.

## Author Contributions

V.X.H., S.P. and R.C.D. conceptualised and designed the experiments. V.X.H., B.A.K., X.L., R.D., H.N., J.H., S.P. performed the experiments and data acquisition. V.X.H. and B.G. performed bioinformatic analysis. V.X.H., S.P., S.S.M., R.C.D. analysed and interpreted the data. V.X.H. and R.C.D. wrote the original manuscript. All authors critically revised and edited the manuscript. All authors approved the final version of the article.

## Conflicts of Interest

The authors declare no conflicts of interest.

## Supporting information


**Figure S1:** Single‐cell RNA sequencing of Sydenham chorea versus controls.
**Figure S2:** Single cell RNA sequencing in Sydenham chorea versus control: Dot plot of significant GO pathways across cell types.
**Methods S1:** Single cell RNA sequencing.

## Data Availability

The data that support the findings of this study are available from the corresponding author upon reasonable request.
