# Supplementary material for: Single‐Cell RNA Seq in Sydenham Chorea Shows B Cell HLA‐DR/DQ Upregulation and Plasma Cell Proteasomal Activation
Source: Ann Clin Transl Neurol. 2025 Aug 26;12(11):2367–71. doi: 10.1002/acn3.70179 (PMC12623827; doi:10.1002/acn3.70179)
Supplement: Supplementary file 1 — Figure S1: Single‐cell RNA sequencing of Sydenham chorea versus controls. Figure S2: Single cell RNA sequencing in Sydenham chorea versus control: Dot plot of significant GO pathways across cell types. Methods S1: Single cell RNA sequencing. [file ACN3-12-2367-s001.pptx]

## Slide 1
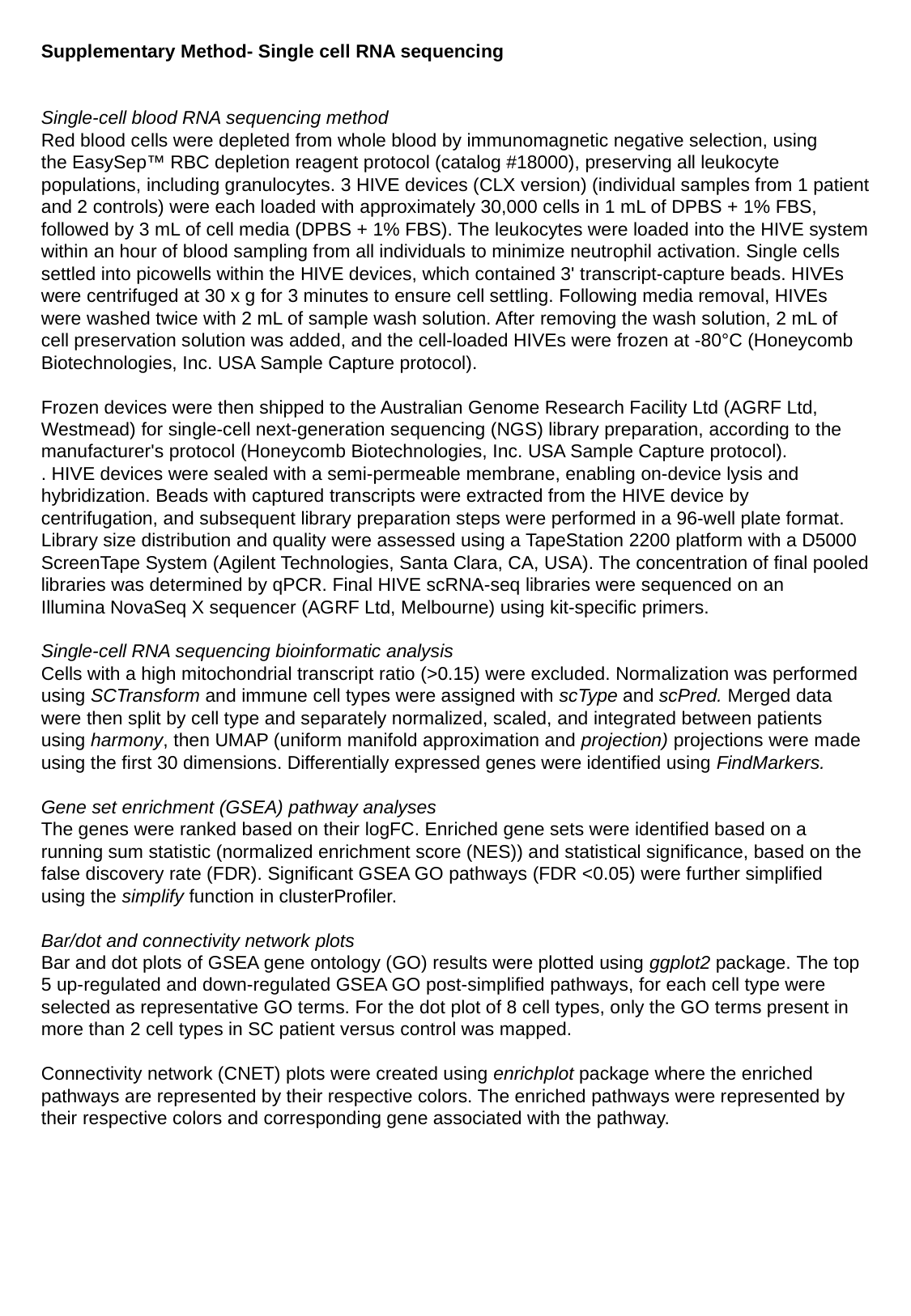

Supplementary Method- Single cell RNA sequencing
Single-cell blood RNA sequencing method
Red blood cells were depleted from whole blood by immunomagnetic negative selection, using the EasySep™ RBC depletion reagent protocol (catalog #18000), preserving all leukocyte populations, including granulocytes. 3 HIVE devices (CLX version) (individual samples from 1 patient and 2 controls) were each loaded with approximately 30,000 cells in 1 mL of DPBS + 1% FBS, followed by 3 mL of cell media (DPBS + 1% FBS). The leukocytes were loaded into the HIVE system within an hour of blood sampling from all individuals to minimize neutrophil activation. Single cells settled into picowells within the HIVE devices, which contained 3' transcript-capture beads. HIVEs were centrifuged at 30 x g for 3 minutes to ensure cell settling. Following media removal, HIVEs were washed twice with 2 mL of sample wash solution. After removing the wash solution, 2 mL of cell preservation solution was added, and the cell-loaded HIVEs were frozen at -80°C (Honeycomb Biotechnologies, Inc. USA Sample Capture protocol).
Frozen devices were then shipped to the Australian Genome Research Facility Ltd (AGRF Ltd, Westmead) for single-cell next-generation sequencing (NGS) library preparation, according to the manufacturer's protocol (Honeycomb Biotechnologies, Inc. USA Sample Capture protocol).
. HIVE devices were sealed with a semi-permeable membrane, enabling on-device lysis and hybridization. Beads with captured transcripts were extracted from the HIVE device by centrifugation, and subsequent library preparation steps were performed in a 96-well plate format. Library size distribution and quality were assessed using a TapeStation 2200 platform with a D5000 ScreenTape System (Agilent Technologies, Santa Clara, CA, USA). The concentration of final pooled libraries was determined by qPCR. Final HIVE scRNA-seq libraries were sequenced on an Illumina NovaSeq X sequencer (AGRF Ltd, Melbourne) using kit-specific primers.
Single-cell RNA sequencing bioinformatic analysis
Cells with a high mitochondrial transcript ratio (>0.15) were excluded. Normalization was performed using SCTransform and immune cell types were assigned with scType and scPred. Merged data were then split by cell type and separately normalized, scaled, and integrated between patients using harmony, then UMAP (uniform manifold approximation and projection) projections were made using the first 30 dimensions. Differentially expressed genes were identified using FindMarkers.
Gene set enrichment (GSEA) pathway analyses
The genes were ranked based on their logFC. Enriched gene sets were identified based on a running sum statistic (normalized enrichment score (NES)) and statistical significance, based on the false discovery rate (FDR). Significant GSEA GO pathways (FDR <0.05) were further simplified using the simplify function in clusterProfiler.
Bar/dot and connectivity network plots
Bar and dot plots of GSEA gene ontology (GO) results were plotted using ggplot2 package. The top 5 up-regulated and down-regulated GSEA GO post-simplified pathways, for each cell type were selected as representative GO terms. For the dot plot of 8 cell types, only the GO terms present in more than 2 cell types in SC patient versus control was mapped.
Connectivity network (CNET) plots were created using enrichplot package where the enriched pathways are represented by their respective colors. The enriched pathways were represented by their respective colors and corresponding gene associated with the pathway.

## Slide 2
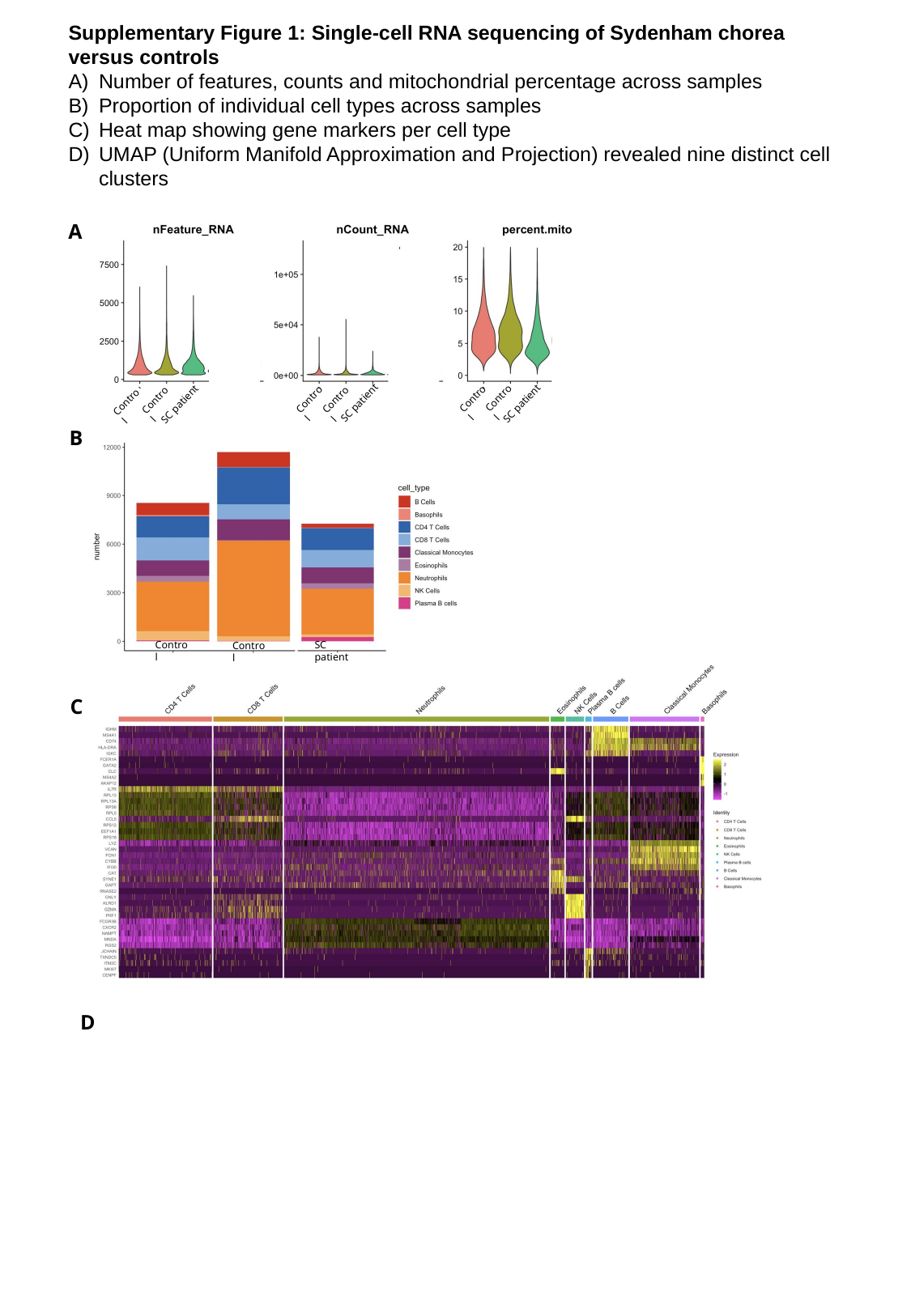

Supplementary Figure 1: Single-cell RNA sequencing of Sydenham chorea versus controls
Number of features, counts and mitochondrial percentage across samples
Proportion of individual cell types across samples
Heat map showing gene markers per cell type
UMAP (Uniform Manifold Approximation and Projection) revealed nine distinct cell clusters
A
Control
Control
Control
Control
Control
SC patient
SC patient
Control
SC patient
B
Control
SC patient
Control
C
D

## Slide 3
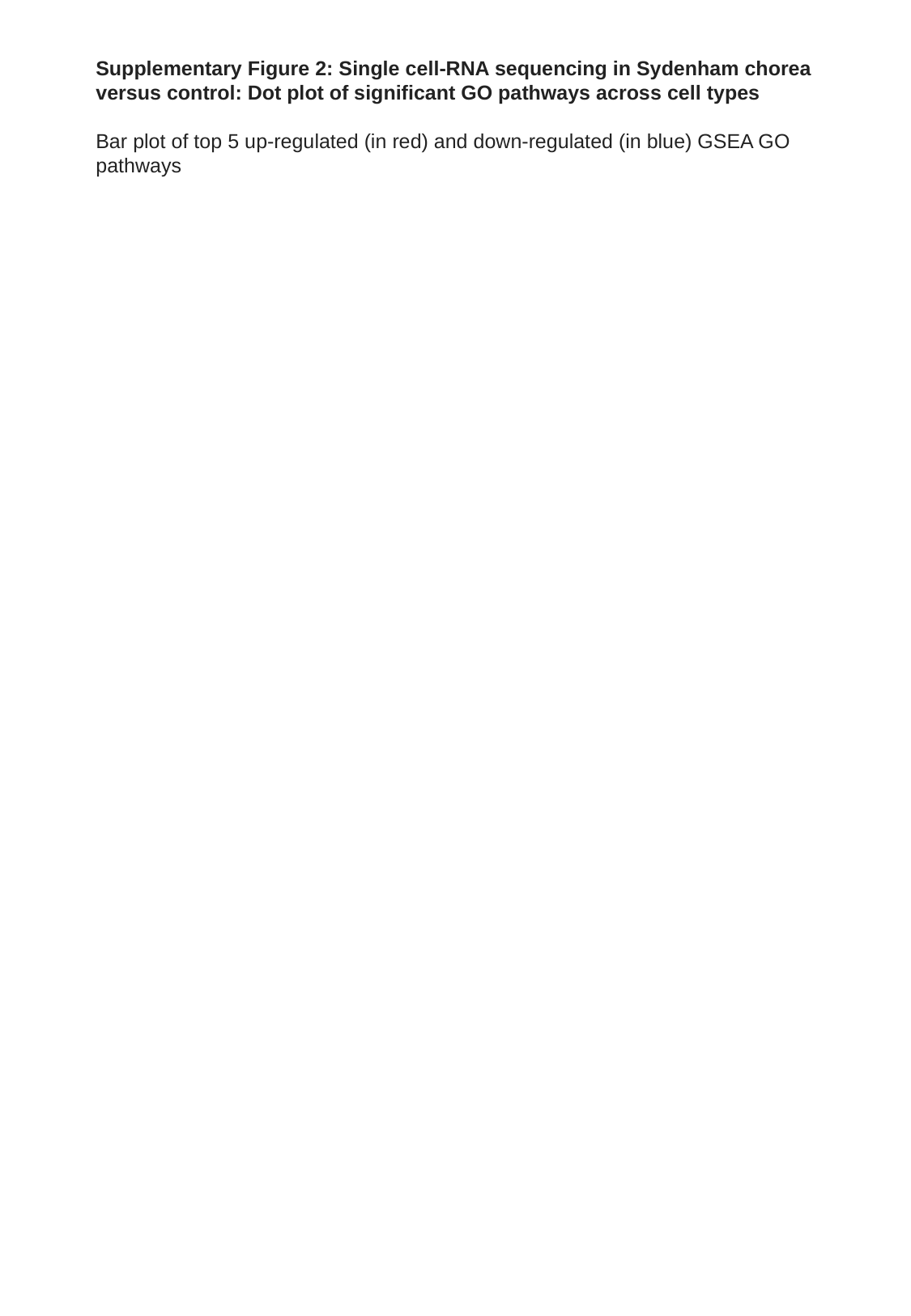

Supplementary Figure 2: Single cell-RNA sequencing in Sydenham chorea versus control: Dot plot of significant GO pathways across cell types
Bar plot of top 5 up-regulated (in red) and down-regulated (in blue) GSEA GO pathways
